# Efficacy of different protein descriptors in predicting protein functional families

**DOI:** 10.1186/1471-2105-8-300

**Published:** 2007-08-17

**Authors:** Serene AK Ong, Hong Huang Lin, Yu Zong Chen, Ze Rong Li, Zhiwei Cao

**Affiliations:** 1 Department of Pharmacy, National University of Singapore, Blk S16, Level 8, 08-14, 3 Science Drive 2, Singapore 117543, Singapore; 2 College of Chemistry, Sichuan University, Chengdu, 610064, P.R. China; 3 Shanghai Center for Bioinformatics Technology, 100, Qinzhou Road, Shanghai 200235 P.R. China

## Abstract

**Background:**

Sequence-derived structural and physicochemical descriptors have frequently been used in machine learning prediction of protein functional families, thus there is a need to comparatively evaluate the effectiveness of these descriptor-sets by using the same method and parameter optimization algorithm, and to examine whether the combined use of these descriptor-sets help to improve predictive performance. Six individual descriptor-sets and four combination-sets were evaluated in support vector machines (SVM) prediction of six protein functional families.

**Results:**

The performance of these descriptor-sets were ranked by Matthews correlation coefficient (MCC), and categorized into two groups based on their performance. While there is no overwhelmingly favourable choice of descriptor-sets, certain trends were found. The combination-sets tend to give slightly but consistently higher MCC values and thus overall best performance such that three out of four combination-sets show slightly better performance compared to one out of six individual descriptor-sets.

**Conclusion:**

Our study suggests that currently used descriptor-sets are generally useful for classifying proteins and the prediction performance may be enhanced by exploring combinations of descriptors.

## Background

Sequence-derived structural and physicochemical descriptors have frequently been used in machine learning prediction of protein structural and functional classes [1-5], protein-protein interactions [6-9], subcellular locations [10-16], peptides containing specific properties[17,18], microarray data [19] and protein secondary structure prediction [20]. These descriptors serve to represent and distinguish proteins or peptides of different structural, functional and interaction profiles by exploring their distinguished features in compositions, correlations, and distributions of the constituent amino acids and their structural and physicochemical properties [2,8,21,22]. There is thus a need to comparatively evaluate the effectiveness of these descriptor-sets for predicting different functional problems by using the same machine learning method and parameter optimization algorithm. Moreover, it is of interest to examine whether combined use of these descriptor-sets help to improve predictive performance.

This work is intended to evaluate the effectiveness of a total of six individual descriptor-sets and four combination-sets (Table 1) in the prediction of several protein functional families by using support vector machine (SVM). Six sets of individual descriptors and three combination-sets have been separately utilized in machine learning prediction of different protein functional and structural properties, all of which have shown impressive predictive performances [22-24]. The six individual sets are amino acid compositions [23] (Set D1), dipeptide compositions [24] (Set D2), normalized Moreau-Broto autocorrelation [25,26] (Set D3), Moran autocorrelation [27] (Set D4), Geary autocorrelation [28] (Set D5), and the composition, transition and distribution of structural and physicochemical properties [2-6,8,17,29,30] (Set D6). The three combination-sets are quasi sequence order formed by weighted sums of amino acid compositions and physicochemical coupling correlations [10,11,18,31] (Set D7), pseudo amino acid composition (PseAA) formed by weighted sums of amino acid compositions and physicochemical square correlations [23,32] (Set D8), and combination of amino acid compositions and dipeptide compositions (Set D9) [24,33]. In this work, we also considered a fourth combination-set that combines descriptor-sets D1 through D8 (Set D10).

**Table 1:** Protein descriptors commonly used for predicting protein functional families.

**Sets**	**Descriptor-sets**	**No. of descriptors (properties)**	**No. of components**	**Type**	**Physicochemical properties**	**Refs**
D1	Amino acid composition	1	20	Sequence composition		[23]
D2	Dipeptide composition	1	400	Sequence composition		[24]
D3	Normalized Moreau – Broto autocorrelation	8	240	Correlation of physicochemical properties	Hydrophobicity scale, average flexibility index, polarizability parameter, free energy of amino acid solution in water, residue accessible surface area, amino acid residue volume, steric parameters, relative mutability	[25, 26]
D4	Moran autocorrelation	8	240	Correlation of physicochemical properties	Hydrophobicity scale, average flexibility index, polarizability parameter, free energy of amino acid solution in water, residue accessible surface area, amino acid residue volume, steric parameters, relative mutability	[27]
D5	Geary autocorrelation	8	240	Square correlation of physicochemical properties	Hydrophobicity scale, average flexibility index, polarizability parameter, free energy of amino acid solution in water, residue accessible surface area, amino acid residue volume, steric parameters, relative mutability	[28]
D6	Descriptors of composition, transition and distribution	21	147	Distribution and variation of physicochemical properties	Hydrophobicity, Van der Waals volume, polarity, polarizability, charge, secondary structures, solvent accessibility	[2-6, 8, 17, 29, 30]
D7	Quasi sequence order	4	160	Combination of sequence composition and correlation of physicochemical	Hydrophobicity, hydrophilicity, polarity, side-chain volume	[10, 11, 18, 31]
D8	Pseudo amino acid composition	3	298	Combination of sequence composition and square correlation of physicochemical	Hydrophobicity, hydrophilicity, side chain mass	[23, 32]
D9	Combination of amino acid and dipeptide composition	2	420	Combination of sequence compositions		
D10	Combination of all eight sets of descriptors	54	1745	Combination of all sets		

The protein functional families studied here include enzyme EC2.4 [34-37], G protein-coupled receptors [38-40], transporter TC8.A [41], chlorophyll [42], lipid synthesis proteins involved in lipid synthesis [43], and rRNA-binding proteins. These six protein families were selected for testing the descriptor-sets based on their functional diversity, sample size and the range of reported family member prediction accuracies [2]. The reported prediction accuracies for these families are generally lower than those of other families [3], which are ideal for critically evaluating the effectiveness of these descriptor-sets; having a lower accuracy should enable a better differentiation of the performance of the various classes. SVM was used as the machine learning method for predicting these functional families because it is a popular method that has consistently been shown better performances than other machine learning methods [44,45]. As this work is intended as a benchmarking study of the performance of various classes of descriptors, other than automatic optimization of results that is an integral part of the SVM programs, such as sigma value scanning, no further attempt was made to optimize the prediction performance of any descriptor class or of any dataset by manually tuning the parameters. Hence, prediction results reported in this paper might differ from those of reported studies.

EC2.4 includes glycosyltransferases that catalyze the synthesis of glycoconjugates and are involved in post-translational modification of proteins (glycosylation). Increased levels of glycosyltransferases have been found in disease states and inflammation [46,47]. TC8.A consists of auxiliary transport proteins that facilitate transport across membranes, which play regulatory and structural roles [48]. GPCR represents G-protein coupled receptors that transduct signals for inducing cellular responses, and members of GPCR are of great pharmacological importance, as 50–60% of approved drugs elicit their therapeutic effect by selectively addressing members of the GPCR family [49-52]. Chlorophyll proteins are essential for harvesting solar energy in photosynthetic antenna systems [53]. Lipid synthesis proteins play central roles in such processes as metabolism, and deficiencies or altered functioning of lipid binding proteins are associated with disease states such as obesity, diabetes, atherosclerosis, hyperlipidemia and insulin resistance [54]. rRNA-binding proteins play central roles in the post-transcriptional regulation of gene expression [55,56], and their binding capabilities are mediated by certain RNA binding domains and motifs [57-60].

## Results and Discussion

The statistics of the six datasets are given in Table 2. Training and prediction statistics for each of the studied descriptor-sets are given in Table 3. Independent validation datasets were used to test the prediction accuracies. Among the 5-fold cross-validation test, independent dataset test and jackknife test, the jackknife is deemed the most rigorous [61]; however, it would have taken a lot of time to use SVM to conduct the jackknife test, thus as a compromise, here we adopted the independent dataset test. The program CDHIT [62-64] was used to remove redundancy at both 90% and 70% sequence identity so to avoid bias, subsequently, the datasets are tested again with the independent evaluation sets and the statistics are given in Table 4. It should be emphasized that the performance evaluation for the studied descriptor-sets are based only on the datasets studied in this work and the conclusions from this study might not be readily extended to other datasets.

**Table 2:** Summary of datasets statistics, including size of training, testing and independent evaluation sets, and average sequence length.

	**Total**		**Training**		**Testing**		**Independent testing**		**Average sequence size**
	P	N	P	N	P	N	P	N	
	
EC2.4	3304	14373	1382	5068	1022	5859	900	3446	460
GPCR	2819	21515	1580	7389	717	7333	522	6793	498
TC8.A	229	23096	94	7962	72	7962	63	7172	483
Chlorophyll	999	22997	356	7928	333	7928	310	7141	480
Lipid	2192	11537	850	5779	707	4483	635	1275	312
rRNA	5855	13770	2004	5246	1940	4953	1911	3571	376

**Table 3:** Dataset training statistics and prediction accuracies of six protein functional families. DS refers to descriptor set, where D1 = amino acid composition; D2 = dipeptide composition; D3 = Moreau-Broto autocorrelation; D4 = Moran autocorrelation; D5 = Geary autocorrelation; D6 = composition, transition and distribution descriptors; D7 = quasi sequence order; D8 = pseudo amino acid composition; D9 = combination of D1+D2; and D10 = combination of D1-D8. Predicted results given as TP (true positive), FN (false negative), TN (true negative), FP (false positive), Sen (sensitivity), Spec (specificity), Q (overall accuracy) and MCC (Matthews correlation coefficient).

**Protein family**	**Des-criptor set**	**Training set**		**Testing set**				**Independent evaluation set**							
		
		P	N	P		N		P			N			Q(%)	MCC
						
				TP	FN	TN	FP	TP	FN	Sen(%)	TN	FP	Spec(%)		
EC2.4	D1	1249	2120	1154	1	9065	12	724	176	80.4	3244	202	94.1	91.3	0.74
	D2	1319	2120	1080	5	8806	1	646	154	82.9	3349	97	97.2	94.1	0.80
	D3	1105	1756	1295	4	9166	5	768	132	85.3	3394	52	98.5	95.8	0.87
	D4	1239	2221	1161	4	8701	5	756	144	84.0	3365	81	97.7	94.8	0.84
	D5	1242	2223	1160	2	8690	14	753	147	83.6	3391	55	98.4	95.4	0.85
	D6	1214	2077	1145	45	8846	4	741	159	82.3	3383	63	98.2	94.9	0.84
	D7	1293	2624	1072	39	8295	8	696	204	77.3	3270	176	94.9	91.3	0.73
	D8	1226	3008	1177	1	7918	1	794	106	88.2	3387	59	98.3	96.2	0.88
	D9	1275	2747	1129	0	8177	3	782	118	86.9	3367	79	97.7	95.5	0.86
	D10	1228	3254	1176	0	7672	1	798	102	88.7	3397	49	98.6	96.5	0.89
															
GPCR	D1	1590	7458	1847	1	14166	3	505	17	96.7	6735	58	99.1	99.0	0.93
	D2	564	711	1728	3	14121	5	510	12	97.7	6737	56	99.2	99.1	0.93
	D3	1169	4628	1122	4	10208	1	507	15	97.1	6737	56	99.2	99.0	0.93
	D4	1257	4474	1037	1	10363	0	499	23	95.6	6745	48	99.3	99.0	0.93
	D5	1290	4724	997	8	10113	0	494	28	94.6	6734	59	99.1	98.8	0.91
	D6	757	2060	1536	2	12777	0	503	19	96.3	6742	51	99.2	99.0	0.93
	D7	812	2950	1482	1	11887	0	495	27	94.8	6696	97	98.6	98.3	0.88
	D8	653	2171	1644	0	12550	1	501	21	96.0	6769	24	99.7	99.4	0.95
	D9	1590	7458	693	12	7322	57	512	10	98.1	6735	58	99.1	99.1	0.93
	D10	672	2454	1625	0	12268	0	502	20	96.2	6757	36	99.5	99.2	0.94
															
TC8.A	D1	118	2858	49	0	13121	0	36	27	57.1	1843	2	99.9	98.5	0.73
	D2	116	1100	50	0	14824	0	41	22	65.1	1843	2	99.9	98.7	0.78
	D3	94	7962	53	0	14501	0	42	21	66.7	1842	3	98.6	98.7	0.78
	D4	94	7962	47	0	11250	0	37	26	58.7	1843	2	99.9	98.5	0.74
	D5	94	7962	47	0	11137	0	37	26	58.7	1843	2	99.9	98.5	0.74
	D6	94	7962	64	0	15283	0	44	19	69.8	1843	2	99.9	98.9	0.81
	D7	94	7962	59	0	15045	0	43	20	68.3	1843	2	99.9	98.9	0.80
	D8	103	943	63	0	14981	0	48	15	76.2	1843	2	99.9	99.1	0.85
	D9	114	810	52	0	15114	0	41	22	65.1	1843	2	99.9	98.7	0.78
	D10	102	1068	64	0	14856	0	48	15	76.2	1843	2	99.9	99.1	0.85
															
Chlorophyll	D1	356	7928	166	0	14297	0	182	128	58.7	1587	11	99.3	92.7	0.71
	D2	4S40	934	248	1	7927	1	228	82	73.6	1595	3	99.8	95.6	0.83
	D3	425	603	264	0	15253	0	246	64	79.4	1594	4	99.8	96.4	0.86
	D4	415	574	273	1	15282	0	247	65	79.7	1597	1	99.9	96.6	0.87
	D5	429	615	259	1	15240	1	233	77	75.2	1597	1	99.9	95.9	0.84
	D6	482	946	202	5	14910	0	205	105	66.1	1597	1	99.9	94.4	0.79
	D7	394	3337	210	85	12517	2	178	132	57.4	1597	1	99.9	93.0	0.73
	D8	371	1421	317	1	14435	0	255	55	82.3	1593	5	99.7	96.9	0.88
	D9	399	1273	289	1	14582	1	249	61	80.3	1591	7	99.6	96.4	0.86
	D10	381	1753	307	1	14102	1	251	59	81.0	1594	4	99.8	96.7	0.88
															
Lipid synthesis	D1	849	2026	705	3	8229	7	470	165	74.0	1218	57	95.5	88.4	0.73
	D2	927	2037	629	1	8225	0	512	123	80.6	1259	16	98.6	92.7	0.84
	D3	898	2968	659	0	7294	0	509	126	80.2	1271	4	99.7	93.2	0.84
	D4	968	3227	588	1	7035	0	493	142	77.6	1273	2	99.8	92.5	0.83
	D5	970	3280	586	1	6982	0	491	144	77.3	1260	15	98.8	91.7	0.81
	D6	874	2112	681	2	8149	1	525	110	82.7	1268	7	99.5	93.9	0.86
	D7	863	2415	692	2	7845	2	512	123	80.6	1271	4	99.7	93.4	0.85
	D8	907	1608	615	0	4488	0	498	137	78.4	1268	7	99.5	92.5	0.83
	D9	815	1613	740	2	8638	11	525	110	82.7	1248	27	97.9	92.8	0.84
	D10	865	1640	657	0	4456	0	531	104	83.6	1268	7	99.5	94.2	0.87
															
rRNA binding	D1	548	579	3390	6	9598	22	1824	87	95.5	3511	60	98.3	97.3	0.94
	D2	1133	1225	2811	0	8974	0	1844	67	96.5	3519	52	98.5	97.8	0.95
	D3	1126	1638	2816	2	8560	1	1812	99	94.8	3535	36	99.0	97.5	0.95
	D4	1337	1958	2697	0	8241	0	1783	128	93.3	3484	87	97.6	96.1	0.91
	D5	1372	1976	2572	0	8223	0	1784	127	93.4	3479	92	97.4	96.0	0.91
	D6	921	1208	2971	52	8991	0	1824	87	95.5	3541	30	99.2	97.9	0.95
	D7	878	2743	3040	26	7442	14	1808	103	97.9	3481	90	97.5	96.5	0.92
	D8	810	2245	3143	0	7954	0	1849	62	96.8	3541	30	99.2	98.3	0.96
	D9	810	972	3075	3	9182	2	1848	63	96.7	3526	45	98.7	98.0	0.96
	D10	900	2600	3044	0	7599	0	1858	53	97.2	3547	24	99.3	98.6	0.97

**Table 4:** Dataset statistics and prediction accuracies after homologous sequences removal (HSR) at 90% and 70% identity. DS refers to descriptor set, where D1 = amino acid composition; D2 = dipeptide composition; D3 = Moreau-Broto autocorrelation; D4 = Moran autocorrelation; D5 = Geary autocorrelation; D6 = composition, transition and distribution descriptors; D7 = quasi sequence order; D8 = pseudo amino acid composition; D9 = combination of D1+D2; and D10 = combination of D1-D8. Predicted results given as TP (true positive), FN (false negative), TN (true negative), FP (false positive), Sen (sensitivity), Spec (specificity), Q (overall accuracy) and MCC (Matthews correlation coefficient).

			**Independent evaluation set**							
			
**Protein family**	**% HSR**	**DS**	P			N			Q (%)	MCC
					
			TP	FN	Sen(%)	TN	FP	Spec(%)		
EC2.4	90	D1	552	250	68.8	3235	201	94.2	89.4	0.65
		D2	626	176	78.1	3339	97	97.2	93.6	0.78
		D3	609	193	75.9	3384	52	98.5	94.2	0.80
		D4	603	199	75.2	3355	81	97.6	93.4	0.78
		D5	591	211	73.7	3381	55	98.4	93.7	0.79
		D6	501	301	62.5	3374	62	98.2	91.4	0.70
		D7	545	257	68.0	3261	175	94.9	89.8	0.66
		D8	666	136	83.0	3375	61	98.2	95.4	0.84
		D9	630	172	78.6	3357	79	97.7	94.1	0.80
		D10	670	132	83.5	3388	48	98.6	95.8	0.86
										
	70	D1	459	223	67.3	3193	199	94.1	89.6	0.62
		D2	516	166	75.7	3296	96	97.2	93.6	0.76
		D3	503	179	73.8	3341	51	98.5	94.4	0.78
		D4	495	187	72.6	3311	81	97.6	93.4	0.75
		D5	484	198	71.0	3339	53	98.4	93.8	0.77
		D6	399	283	58.5	3330	62	98.2	91.5	0.67
		D7	452	230	66.3	3218	174	94.9	90.1	0.63
		D8	551	131	80.8	3331	61	98.2	95.3	0.83
		D9	520	162	76.3	3314	78	97.7	94.1	0.78
		D10	554	128	81.2	3344	48	98.6	95.7	0.84

GPCR	90	D1	391	13	96.8	6724	58	99.1	99.0	0.91
		D2	395	9	97.8	6744	38	99.4	99.4	0.94
		D3	393	11	97.3	6726	56	99.2	99.1	0.92
		D4	386	18	95.5	6734	48	99.3	99.1	0.92
		D5	381	23	94.3	6723	59	99.1	98.9	0.90
		D6	391	13	96.8	6731	51	99.3	99.1	0.92
		D7	382	22	94.6	6685	97	98.6	98.3	0.86
		D8	387	17	95.8	6758	24	99.7	99.4	0.95
		D9	391	13	96.8	6752	30	99.6	99.4	0.94
		D10	388	16	96.0	6762	20	99.7	99.5	0.95
										
	70	D1	307	8	97.5	6695	58	99.1	99.1	0.90
		D2	309	6	98.1	6715	38	99.4	99.4	0.93
		D3	306	9	97.1	6697	56	99.2	99.1	0.90
		D4	301	14	95.6	6705	48	99.3	99.1	0.90
		D5	198	17	94.6	6694	59	99.1	98.9	0.88
		D6	307	8	97.5	6702	51	99.2	99.2	0.91
		D7	296	19	94.0	6656	97	98.6	98.4	0.83
		D8	301	14	95.6	6729	24	99.6	99.5	0.94
		D9	307	8	97.5	6723	30	99.6	99.5	0.94
		D10	302	13	95.9	6733	20	99.7	99.5	0.95

TC8.A	90	D1	28	27	50.9	1846	2	99.9	98.5	0.68
		D2	33	22	60.0	1846	2	99.9	98.7	0.75
		D3	34	21	61.8	1845	3	99.8	98.7	0.75
		D4	29	26	52.7	1845	3	99.8	98.8	0.75
		D5	29	26	52.7	1845	3	99.8	98.8	0.75
		D6	36	19	65.5	1846	2	99.9	98.9	0.78
		D7	35	20	63.6	1845	3	99.8	98.8	0.76
		D8	40	15	72.7	1845	3	99.8	99.2	0.82
		D9	33	22	60.0	1846	2	99.9	98.7	0.75
		D10	40	15	72.7	1845	3	99.8	99.2	0.82
										
	70	D1	25	24	51.0	1828	2	99.9	98.6	0.68
		D2	29	20	59.2	1828	2	99.9	98.8	0.74
		D3	29	20	59.2	1827	3	99.8	98.8	0.73
		D4	26	23	53.1	1828	2	99.9	98.7	0.70
		D5	26	23	53.1	1828	2	99.9	98.7	0.70
		D6	33	16	67.3	1828	2	99.9	99.0	0.79
		D7	30	19	61.2	1827	3	99.8	98.8	0.74
		D8	36	13	73.5	1827	3	99.8	99.2	0.82
		D9	29	20	59.2	1828	2	99.9	98.8	0.74
		D10	36	13	73.5	1827	3	99.8	99.2	0.82

Chlorophyll	90	D1	159	127	55.6	1594	8	99.5	92.9	0.70
		D2	205	81	71.7	1598	4	99.8	95.5	0.82
		D3	224	62	78.3	1599	3	99.8	96.6	0.86
		D4	222	64	77.6	1599	3	99.8	96.5	0.86
		D5	211	75	73.8	1598	4	99.8	95.8	0.83
		D6	182	104	63.6	1594	8	99.5	94.1	0.75
		D7	159	127	55.6	1595	9	99.4	92.8	0.69
		D8	233	53	81.5	1595	7	99.6	96.8	0.87
		D9	224	62	78.3	1594	8	99.5	96.3	0.85
		D10	229	57	80.1	1597	5	99.7	96.7	0.87
										
	70	D1	113	118	48.9	1578	8	99.5	93.1	0.65
		D2	155	76	67.1	1582	4	99.8	95.6	0.79
		D3	171	60	74.0	1583	3	99.8	96.5	0.84
		D4	171	60	74.0	1583	3	99.8	96.5	0.84
		D5	161	70	69.7	1582	4	99.8	95.9	0.81
		D6	137	94	59.3	1578	8	99.5	94.4	0.72
		D7	114	117	49.4	1575	11	99.3	93.0	0.64
		D8	182	49	78.8	1579	7	99.6	96.9	0.85
		D9	172	59	74.5	1578	8	99.5	96.3	0.82
		D10	178	53	77.1	1581	5	99.7	96.8	0.85

Lipid synthesis	90	D1	403	149	73.0	1213	59	95.4	88.6	0.72
		D2	431	121	78.1	1256	16	98.7	92.5	0.81
		D3	436	116	79.0	1268	4	99.7	93.4	0.84
		D4	421	131	76.3	1270	2	99.8	92.7	0.83
		D5	416	136	75.4	1270	2	99.8	92.4	0.82
		D6	449	103	81.3	1270	2	99.8	94.2	0.86
		D7	435	117	78.8	1269	3	99.8	93.4	0.84
		D8	423	129	76.6	1265	7	99.5	92.5	0.82
		D9	449	103	81.3	1245	27	97.9	92.9	0.83
		D10	454	98	82.3	1265	7	99.5	94.2	0.86
										
	70	D1	316	138	69.6	1205	59	95.3	88.5	0.69
		D2	343	111	75.6	1248	16	98.7	92.6	0.81
		D3	340	114	74.9	1260	4	99.7	93.1	0.82
		D4	330	124	72.7	1262	2	99.8	92.7	0.81
		D5	328	126	72.3	1260	4	99.7	92.4	0.80
		D6	358	96	78.9	1244	20	98.4	93.3	0.82
		D7	342	112	75.3	1257	7	99.5	93.1	0.82
		D8	331	123	72.9	1257	7	99.4	92.4	0.80
		D9	360	94	79.3	1237	27	97.9	93.0	0.81
		D10	360	94	79.3	1257	7	99.5	94.1	0.85

rRNA binding	90	D1	1407	91	93.9	3502	59	98.3	97.0	0.93
		D2	1437	61	95.9	3510	51	98.6	97.8	0.95
		D3	1403	95	93.7	3529	32	99.1	97.5	0.93
		D4	1347	151	89.9	3491	70	98.0	95.6	0.89
		D5	1347	151	89.9	3533	28	99.2	96.5	0.91
		D6	1451	47	96.9	3537	24	99.3	98.6	0.97
		D7	1358	140	90.7	3429	132	96.3	94.6	0.87
		D8	1442	56	96.3	3531	30	99.2	98.3	0.96
		D9	1436	62	95.9	3518	43	98.8	97.9	0.95
		D10	1449	49	96.7	3537	24	99.3	98.6	0.97
										
	70	D1	924	83	91.8	3454	59	98.3	96.9	0.91
		D2	952	55	94.5	3463	50	98.6	97.7	0.93
		D3	920	87	91.4	3483	30	99.2	97.4	0.92
		D4	907	100	90.1	3444	69	98.0	96.3	0.89
		D5	908	99	90.2	3485	28	99.2	97.2	0.92
		D6	963	44	95.6	3493	20	99.4	98.6	0.96
		D7	917	90	91.1	3382	131	96.3	95.1	0.86
		D8	654	53	94.7	3484	29	99.2	98.2	0.95
		D9	950	57	94.3	3471	42	98.8	97.8	0.94
		D10	960	47	95.3	3490	23	99.4	98.5	0.96

The performance of the ten descriptor-sets were ranked by the Matthews correlation coefficient (MCC) values of the respective SVM prediction of the six functional families, which are given in Table 5. The computed MCC scores for these descriptor-sets are in the range of 0.64~0.97 for all protein families studied. Accordingly, the performance of these descriptor-sets is categorized into two groups based on their MCC values: 'Exceptional' (>0.85) and 'Good' (≤0.85). Moreover, these descriptor-sets are aligned in the order of their MCC values with "=" being of equal values and ">" indicating that one is better than the other. It is noted that, as the differences of many of these MCC values are rather small, such alignment is likely superficial to some extent and may not best reflect the real ranking of performance. Overall, the performances of these descriptor-sets are not significantly different, there is no overwhelmingly preferred descriptor-set, and SVM prediction performance appears to be highly dependent on the dataset.

**Table 5:** Descriptor sets ranked and grouped by MCC (Matthews correlation coefficient), before and after removal of homologous sequences at 90% and 70% identity, respectively.

**Protein family**	% HRS*	Prediction performance	
		
		**Exceptional **> 0.85	**Good **= 0.85
EC2.4	NR	D10 > D8> D9 > D3	D5 > D4 = D6 > D2 > D1 > D7
	90%	D10	D8 > D3 = D9 > D5 > D2 = D4 > D6 > D7 > D1
	70%		D10 > D8 > D3 = D9 > D5 > D2 > D4 > D6 > D7 > D1
GPCR	NR	D8 > D10 > D1 = D2 = D3 = D4 = D6 = D9 > D5 > D7	
	90%	D8 = D10 > D2 = D9 > D3 = D4 = D6 > D1 > D5 > D7	
	70%	D10 > D8 = D9 > D2 > D6 > D1 = D3 = D4 > D5	D7
TC8.A	NR		D8 = D10 > D6 > D7 > D2 = D3 = D9 > D4 = D5 > D1
	90%		D8 = D10 > D6 > D7 > D2 = D3 = D4 = D5 = D9 > D1
	70%		D8 = D10 > D6 > D2 = D7 = D9 > D3 > D4 = D5 > D1
Chlorophyll	NR	D8 = D10 > D4 > D3 = D9	D5 > D2 > D6 > D7 > D1
	90%	D8 = D10 > D3 = D4	D9 > D5 > D2 > D6 > D1 > D7
	70%		D8 = D10 > D3 = D4 > D9 > D5 > D2 > D6 > D1 > D7
Lipid synthesis	NR	D10 > D6	D7 > D2 = D3 = D9 > D4 = D8 > D5 > D1
	90%	D6 = D10	D3 = D7 > D4 = D9 > D5 = D8 > D2 > D1
	70%		D10 > D3 = D6 = D7 > D2 = D4 = D9 > D5 = D8 > D1
rRNA binding	NR	D10 > D8 = D9 > D2 = D3 = D6 > D1 > D7> D4 = D5	
	90%	D6 = D10 > D8 > D2 = D9 > D1 = D3 > D5 > D4> D7	
	70%	D6 = D10> D8 > D9 > D2 > D3 = D5 > D1 > D4 > D7	

*HSR: homologous sequence removed

NR: (homologous sequences) Not Removed

As shown in Table 3 and Table 4, for many of the studied datasets, the differences in prediction accuracies and MCC values between different descriptor-sets are small. In particular, for GPCR and rRNA binding proteins, the results of almost all descriptor-sets are in the 'Exceptional' category. Examining the range of MCC values of the descriptor-sets for each of the studied protein families (after removal of 70% homologous sequences), the differences between the largest and smallest MCC values are, in order of increasing magnitude: 0.10, 0.12, 0.14, 0.16, 0.21 and 0.21 for rRNA binding proteins, GPCR, TC8.A, lipid synthesis proteins, chlorophyll proteins and EC.2.4 families respectively. Given that a difference of 0.10 and 0.20 in MCC values translates to an approximate 4% and 7% difference in overall prediction accuracy, this separation is not large indeed.

Though the dataset is a more important determinant of prediction performance than the choice of descriptor class, a few general trends could be observed. Three out of four of the combination-sets tend to exhibit slightly but consistently higher MCC values for the protein families studied in this work. These sets are Sets D8, D9 and D10. In contrast, only one out of six individual sets, Set D6, tend to exhibit slightly but consistently higher MCC values for the protein families studied in this work. Therefore, statistically speaking, it appears that the use of combination-sets tend to give slightly better prediction performance than the use of individual-sets.

When each class was examined individually in this study, we find that the combination of amino acid composition and dipeptide composition (Set D9) tends to give consistently better results than that of the individual descriptor-sets (Set D1 and Set D2). It has been reported that one drawback of amino acid composition descriptors is that the same amino acid composition may correspond to diverse sequences as sequence order is lost [24,33]. This sequence order information can be partially covered by considering dipeptide composition (Set D2). On the other hand, dipeptide composition lacks information concerning the fraction of the individual residue in the sequence, thus, a combination-set is expected to give better prediction results [24,33,65,66].

Using all descriptor-sets (Set D10) generally, but not always, gives the best result, which is consistent with the findings on the use of molecular descriptors for predicting compounds of specific properties. [67,68] For instance, Xue *et al*. found that feature selection methods are capable of reducing the noise generated by the use of overlapping and redundant molecular descriptors, and in some cases, improving the accuracy of SVM classification of pharmacokinetic behaviour of chemical agents [69]. In our study, for example, the three autocorrelation descriptor-sets (Sets D3, D4 and D5) all utilize the same physicochemical properties, only differing in the correlation algorithm. The use of all available descriptors likely results in the inclusion of partially redundant information, some of which may to some extent become noise that interferes with the prediction results or obscures relevant information. Based on the results of previous studies [69], it is possible that feature selection methods may be applied for selecting the optimal set of descriptors to improve prediction accuracy as well as computing efficiency for predicting protein functional families.

## Conclusion

The effectiveness of ten protein descriptor-sets in six protein functional family prediction using SVM was evaluated. Corroborating with previous work done on chemical descriptors [67,68,70-76] and protein descriptors [4,21,30,32,35,43,77,78], we found that the descriptor-sets evaluated in this paper, which comprise some of the commonly used descriptors, generally return good results and do not differ significantly. In particular, the use of combination descriptor-sets tends to give slightly better prediction performance than the use of individual descriptor-sets. While there seems to be no preferred descriptor-set that could be utilized for all datasets as prediction results is highly dependent on datasets, the performance of protein classification may be enhanced by selection of optimal combinations of descriptors using established feature-selection methods [79,80]. Incorporation of appropriate sets of physicochemical properties not covered by some of the existing descriptor-sets may also help improving the prediction performance.

## Methods

### Datasets

The datasets were obtained from SwissProt [81], except for TC8.A, which was downloaded from Transport Classification Database (TCDB) [41]. These datasets were chosen for their functional diversity, sample size and the range of reported family member prediction accuracies. As SVM is essentially a statistical method, the datasets cannot be too small; yet it would also be convenient for the purposes of this study if they were not too large as to be unwieldy computationally. These downloaded datasets were used to construct the positive dataset for the corresponding SVM classification system. A negative dataset, representing non-class members, was generated by a well-established procedure [2,3,21,30] such that all proteins was grouped into domain families [82] in the PFAM database, and the representative proteins of these families unrelated to the protein family being studied were chosen as negative samples.

These proteins, positive and negative, were further divided into separate training, testing and independent evaluation sets by the following procedure: First, proteins were converted into descriptor vectors and then clustered using hierarchical clustering into groups in the structural and physicochemical feature space [83], where more homologous sequences will have shorter distances between them, and the largest separation between clusters was set to a ceiling of 20. One representative protein was randomly selected from each group to form a training set that is sufficiently diverse and broadly distributed in the feature space. Another protein within the group was randomly selected to form the testing set. The selected proteins from each group were further checked to ensure that they are distinguished from the proteins in other groups. The remaining proteins were then designated as the independent evaluation set, also checked to be at a reasonable level of diversity. Fragments, defined as smaller than 60 residues, were discarded. This selection process ensures that the training, testing and evaluation sets constructed are sufficiently diverse and broadly distributed in the feature space. Though an analysis of the 'similar' proteins in each cluster showed that the majority of the proteins in a cluster are quite non-homologous, the program CDHIT (Cluster Database at High Identity with Tolerance) [62-64] was further used after the SVM model was trained to remove redundancy at both 90% and 70% sequence identity, so as to avoid bias as far as possible. CDHIT removes homologous sequences by clustering the protein dataset at some user-defined sequence identity threshold, for example 90%, and then generating a database of only the cluster representatives, thus eliminating sequences with greater than 90% identity. The statistical details are given in Tables 2 and 3.

### Algorithms for generating protein descriptors

Ten sets of commonly used composition and physicochemical descriptors were generated from the protein sequence (see Table 1). These descriptors can be computed via the PROFEAT server [22].

Amino acid composition (Set D1) is defined as the fraction of each amino acid type in a sequence

f(r)=NrN,
 MathType@MTEF@5@5@+=feaafiart1ev1aaatCvAUfKttLearuWrP9MDH5MBPbIqV92AaeXatLxBI9gBaebbnrfifHhDYfgasaacH8akY=wiFfYdH8Gipec8Eeeu0xXdbba9frFj0=OqFfea0dXdd9vqai=hGuQ8kuc9pgc9s8qqaq=dirpe0xb9q8qiLsFr0=vr0=vr0dc8meaabaqaciaacaGaaeqabaqabeGadaaakeaacqWGMbGzcqGGOaakcqWGYbGCcqGGPaqkcqGH9aqpdaWcaaqaaiabd6eaonaaBaaaleaacqWGYbGCaeqaaaGcbaGaemOta4eaaiabcYcaSaaa@3703@

where *r *= 1, 2, ..., 20, *N*_*r *_is the number of amino acid of type *r*, and *N *is the length of the sequence. Dipeptide composition (Set D2) is defined as

fr(r,s)=NrsN−1,
 MathType@MTEF@5@5@+=feaafiart1ev1aaatCvAUfKttLearuWrP9MDH5MBPbIqV92AaeXatLxBI9gBaebbnrfifHhDYfgasaacH8akY=wiFfYdH8Gipec8Eeeu0xXdbba9frFj0=OqFfea0dXdd9vqai=hGuQ8kuc9pgc9s8qqaq=dirpe0xb9q8qiLsFr0=vr0=vr0dc8meaabaqaciaacaGaaeqabaqabeGadaaakeaacqWGMbGzcqWGYbGCcqGGOaakcqWGYbGCcqGGSaalcqWGZbWCcqGGPaqkcqGH9aqpdaWcaaqaaiabd6eaonaaBaaaleaacqWGYbGCcqWGZbWCaeqaaaGcbaGaemOta4KaeyOeI0IaeGymaedaaiabcYcaSaaa@3E0B@

where *r*, *s *= 1, 2, ..., 20, *N*_*ij *_is the number of dipeptides composed of amino acid types *r *and *s*.

Autocorrelation descriptors are a class of topological descriptors, also known as molecular connectivity indices, describe the level of correlation between two objects (protein or peptide sequences) in terms of their specific structural or physicochemical property [84], which are defined based on the distribution of amino acid properties along the sequence [85]. Eight amino acid properties are used for deriving the autocorrelation descriptors: hydrophobicity scale [86]; average flexibility index [87]; polarizability parameter [88]; free energy of amino acid solution in water [88]; residue accessible surface areas [89]; amino acid residue volumes [90]; steric parameters [91]; and relative mutability [92].

These autocorrelation properties are normalized and standardized such that

Pr'=Pr−p¯σ,
 MathType@MTEF@5@5@+=feaafiart1ev1aaatCvAUfKttLearuWrP9MDH5MBPbIqV92AaeXatLxBI9gBaebbnrfifHhDYfgasaacH8akY=wiFfYdH8Gipec8Eeeu0xXdbba9frFj0=OqFfea0dXdd9vqai=hGuQ8kuc9pgc9s8qqaq=dirpe0xb9q8qiLsFr0=vr0=vr0dc8meaabaqaciaacaGaaeqabaqabeGadaaakeaacqWGqbaudaqhaaWcbaGaemOCaihabaGaei4jaCcaaOGaeyypa0ZaaSaaaeaacqWGqbaudaWgaaWcbaGaemOCaihabeaakiabgkHiTiqbdchaWzaaraaabaacciGae83WdmhaaiabcYcaSaaa@3949@

where P¯
 MathType@MTEF@5@5@+=feaafiart1ev1aaatCvAUfKttLearuWrP9MDH5MBPbIqV92AaeXatLxBI9gBaebbnrfifHhDYfgasaacH8akY=wiFfYdH8Gipec8Eeeu0xXdbba9frFj0=OqFfea0dXdd9vqai=hGuQ8kuc9pgc9s8qqaq=dirpe0xb9q8qiLsFr0=vr0=vr0dc8meaabaqaciaacaGaaeqabaqabeGadaaakeaacuWGqbaugaqeaaaa@2DED@ is the average value of a particular property of the 20 amino acids. P¯
 MathType@MTEF@5@5@+=feaafiart1ev1aaatCvAUfKttLearuWrP9MDH5MBPbIqV92AaeXatLxBI9gBaebbnrfifHhDYfgasaacH8akY=wiFfYdH8Gipec8Eeeu0xXdbba9frFj0=OqFfea0dXdd9vqai=hGuQ8kuc9pgc9s8qqaq=dirpe0xb9q8qiLsFr0=vr0=vr0dc8meaabaqaciaacaGaaeqabaqabeGadaaakeaacuWGqbaugaqeaaaa@2DED@ and σ are given by

P¯=∑r=120Pr20,
 MathType@MTEF@5@5@+=feaafiart1ev1aaatCvAUfKttLearuWrP9MDH5MBPbIqV92AaeXatLxBI9gBaebbnrfifHhDYfgasaacH8akY=wiFfYdH8Gipec8Eeeu0xXdbba9frFj0=OqFfea0dXdd9vqai=hGuQ8kuc9pgc9s8qqaq=dirpe0xb9q8qiLsFr0=vr0=vr0dc8meaabaqaciaacaGaaeqabaqabeGadaaakeaacuWGqbaugaqeaiabg2da9maalaaabaWaaabCaeaacqWGqbaudaWgaaWcbaGaemOCaihabeaaaeaacqWGYbGCcqGH9aqpcqaIXaqmaeaacqaIYaGmcqaIWaama0GaeyyeIuoaaOqaaiabikdaYiabicdaWaaacqGGSaalaaa@3C09@

and

σ=120∑r=120(Pr−P¯)2.
 MathType@MTEF@5@5@+=feaafiart1ev1aaatCvAUfKttLearuWrP9MDH5MBPbIqV92AaeXatLxBI9gBaebbnrfifHhDYfgasaacH8akY=wiFfYdH8Gipec8Eeeu0xXdbba9frFj0=OqFfea0dXdd9vqai=hGuQ8kuc9pgc9s8qqaq=dirpe0xb9q8qiLsFr0=vr0=vr0dc8meaabaqaciaacaGaaeqabaqabeGadaaakeaaiiGacqWFdpWCcqGH9aqpdaGcaaqaamaalaaabaGaeGymaedabaGaeGOmaiJaeGimaadaamaaqahabaGaeiikaGIaemiuaa1aaSbaaSqaaiabdkhaYbqabaGccqGHsislcuWGqbaugaqeaiabcMcaPmaaCaaaleqabaGaeGOmaidaaaqaaiabdkhaYjabg2da9iabigdaXaqaaiabikdaYiabicdaWaqdcqGHris5aaWcbeaakiabc6caUaaa@42AA@

Moreau-Broto autocorrelation descriptors (Set D3) [84,93] are defined as

AC(d)=∑i=1N−dPiPi+d,
 MathType@MTEF@5@5@+=feaafiart1ev1aaatCvAUfKttLearuWrP9MDH5MBPbIqV92AaeXatLxBI9gBaebbnrfifHhDYfgasaacH8akY=wiFfYdH8Gipec8Eeeu0xXdbba9frFj0=OqFfea0dXdd9vqai=hGuQ8kuc9pgc9s8qqaq=dirpe0xb9q8qiLsFr0=vr0=vr0dc8meaabaqaciaacaGaaeqabaqabeGadaaakeaacqWGbbqqcqWGdbWqcqGGOaakcqWGKbazcqGGPaqkcqGH9aqpdaaeWbqaaiabdcfaqnaaBaaaleaacqWGPbqAaeqaaOGaemiuaa1aaSbaaSqaaiabdMgaPjabgUcaRiabdsgaKbqabaaabaGaemyAaKMaeyypa0JaeGymaedabaGaemOta4KaeyOeI0IaemizaqganiabggHiLdGccqGGSaalaaa@4441@

where *d *= 1, 2, ..., 30 is the lag of the autocorrelation, and *P*_*i *_and *P*_*i*+*d *_are the properties of the amino acid at positions *i *and *i+d *respectively. After applying normalization, we get

ATS(d)=AC(d)N−d.
 MathType@MTEF@5@5@+=feaafiart1ev1aaatCvAUfKttLearuWrP9MDH5MBPbIqV92AaeXatLxBI9gBaebbnrfifHhDYfgasaacH8akY=wiFfYdH8Gipec8Eeeu0xXdbba9frFj0=OqFfea0dXdd9vqai=hGuQ8kuc9pgc9s8qqaq=dirpe0xb9q8qiLsFr0=vr0=vr0dc8meaabaqaciaacaGaaeqabaqabeGadaaakeaacqWGbbqqcqWGubavcqWGtbWucqGGOaakcqWGKbazcqGGPaqkcqGH9aqpdaWcaaqaaiabdgeabjabdoeadjabcIcaOiabdsgaKjabcMcaPaqaaiabd6eaojabgkHiTiabdsgaKbaacqGGUaGlaaa@3D94@

Moran autocorrelation descriptors (Set D4) [94] are calculated as

I(d)=1N−d∑i=1N−d(Pi−P¯)(Pi+d−P¯)1N∑i=1N(Pi−P¯)2,
 MathType@MTEF@5@5@+=feaafiart1ev1aaatCvAUfKttLearuWrP9MDH5MBPbIqV92AaeXatLxBI9gBaebbnrfifHhDYfgasaacH8akY=wiFfYdH8Gipec8Eeeu0xXdbba9frFj0=OqFfea0dXdd9vqai=hGuQ8kuc9pgc9s8qqaq=dirpe0xb9q8qiLsFr0=vr0=vr0dc8meaabaqaciaacaGaaeqabaqabeGadaaakeaacqWGjbqscqGGOaakcqWGKbazcqGGPaqkcqGH9aqpdaWcaaqaamaalaaabaGaeGymaedabaGaemOta4KaeyOeI0IaemizaqgaamaaqahabaGaeiikaGIaemiuaa1aaSbaaSqaaiabdMgaPbqabaGccqGHsislcuWGqbaugaqeaiabcMcaPiabcIcaOiabdcfaqnaaBaaaleaacqWGPbqAcqGHRaWkcqWGKbazaeqaaOGaeyOeI0IafmiuaaLbaebacqGGPaqkaSqaaiabdMgaPjabg2da9iabigdaXaqaaiabd6eaojabgkHiTiabdsgaKbqdcqGHris5aaGcbaWaaSaaaeaacqaIXaqmaeaacqWGobGtaaWaaabCaeaacqGGOaakcqWGqbaudaWgaaWcbaGaemyAaKgabeaakiabgkHiTiqbdcfaqzaaraGaeiykaKYaaWbaaSqabeaacqaIYaGmaaaabaGaemyAaKMaeyypa0JaeGymaedabaGaemOta4eaniabggHiLdaaaOGaeiilaWcaaa@601F@

where *d*, *P*_*i *_and *P*_*i*+*d *_are defined in the same way as that for Moreau-Broto autocorrelation and P¯
 MathType@MTEF@5@5@+=feaafiart1ev1aaatCvAUfKttLearuWrP9MDH5MBPbIqV92AaeXatLxBI9gBaebbnrfifHhDYfgasaacH8akY=wiFfYdH8Gipec8Eeeu0xXdbba9frFj0=OqFfea0dXdd9vqai=hGuQ8kuc9pgc9s8qqaq=dirpe0xb9q8qiLsFr0=vr0=vr0dc8meaabaqaciaacaGaaeqabaqabeGadaaakeaacuWGqbaugaqeaaaa@2DED@ is the average of the considered property *P *along the sequence:

P¯=∑i=1NPiN.
 MathType@MTEF@5@5@+=feaafiart1ev1aaatCvAUfKttLearuWrP9MDH5MBPbIqV92AaeXatLxBI9gBaebbnrfifHhDYfgasaacH8akY=wiFfYdH8Gipec8Eeeu0xXdbba9frFj0=OqFfea0dXdd9vqai=hGuQ8kuc9pgc9s8qqaq=dirpe0xb9q8qiLsFr0=vr0=vr0dc8meaabaqaciaacaGaaeqabaqabeGadaaakeaacuWGqbaugaqeaiabg2da9maalaaabaWaaabCaeaacqWGqbaudaWgaaWcbaGaemyAaKgabeaaaeaacqWGPbqAcqGH9aqpcqaIXaqmaeaacqWGobGta0GaeyyeIuoaaOqaaiabd6eaobaacqGGUaGlaaa@3A73@

Geary autocorrelation descriptors (Set D5) [95] are written as

C(d)=12(N−d)∑i=1N−d(Pi−Pi+d)21N−1∑i=1N(Pi−P¯)2,
 MathType@MTEF@5@5@+=feaafiart1ev1aaatCvAUfKttLearuWrP9MDH5MBPbIqV92AaeXatLxBI9gBaebbnrfifHhDYfgasaacH8akY=wiFfYdH8Gipec8Eeeu0xXdbba9frFj0=OqFfea0dXdd9vqai=hGuQ8kuc9pgc9s8qqaq=dirpe0xb9q8qiLsFr0=vr0=vr0dc8meaabaqaciaacaGaaeqabaqabeGadaaakeaacqWGdbWqcqGGOaakcqWGKbazcqGGPaqkcqGH9aqpdaWcaaqaamaalaaabaGaeGymaedabaGaeGOmaiJaeiikaGIaemOta4KaeyOeI0IaemizaqMaeiykaKcaamaaqahabaGaeiikaGIaemiuaa1aaSbaaSqaaiabdMgaPbqabaGccqGHsislcqWGqbaudaWgaaWcbaGaemyAaKMaey4kaSIaemizaqgabeaakiabcMcaPmaaCaaaleqabaGaeGOmaidaaaqaaiabdMgaPjabg2da9iabigdaXaqaaiabd6eaojabgkHiTiabdsgaKbqdcqGHris5aaGcbaWaaSaaaeaacqaIXaqmaeaacqWGobGtcqGHsislcqaIXaqmaaWaaabCaeaacqGGOaakcqWGqbaudaWgaaWcbaGaemyAaKgabeaakiabgkHiTiqbdcfaqzaaraGaeiykaKYaaWbaaSqabeaacqaIYaGmaaaabaGaemyAaKMaeyypa0JaeGymaedabaGaemOta4eaniabggHiLdaaaOGaeiilaWcaaa@6087@

where *d*, P¯
 MathType@MTEF@5@5@+=feaafiart1ev1aaatCvAUfKttLearuWrP9MDH5MBPbIqV92AaeXatLxBI9gBaebbnrfifHhDYfgasaacH8akY=wiFfYdH8Gipec8Eeeu0xXdbba9frFj0=OqFfea0dXdd9vqai=hGuQ8kuc9pgc9s8qqaq=dirpe0xb9q8qiLsFr0=vr0=vr0dc8meaabaqaciaacaGaaeqabaqabeGadaaakeaacuWGqbaugaqeaaaa@2DED@, *P*_*i *_and *P*_*i*+*d *_are defined as above. Comparing the three autocorrelation descriptors: while Moreau-Broto autocorrelation uses the property values as the basis for measurement, Moran autocorrelation utilizes property deviations from the average values, and Geary utilizes the square-difference of property values instead of vector-products (of property values or deviations). The Moran and Geary autocorrelation descriptors measure spatial autocorrelation, which is the correlation of a variable with itself through space.

The descriptors in Set D6 comprise of the composition (*C*), transition (*T*) and distribution (*D*) features of seven structural or physicochemical properties along a protein or peptide sequence [5,29]. The seven physicochemical properties [2,5,29] are hydrophobicity; normalized Van der Waals volume; polarity; polarizibility; charge; secondary structures; and solvent accessibility. For each of these properties, the amino acids are divided into three groups such that those in a particular group are regarded to have approximately the same property. For instance, residues can be divided into hydrophobic (CVLIMFW), neutral (GASTPHY), and polar (RKEDQN) groups. *C *is defined as the number of residues with that particular property divided by the total number of residues in a protein sequence. *T *characterizes the percent frequency with which residues with a particular property is followed by residues of a different property. *D *measures the chain length within which the first, 25%, 50%, 75% and 100% of the amino acids with a particular property are located respectively. There are 21 elements representing these three descriptors: 3 for *C*, 3 for *T *and 15 for *D*, and the protein feature vector is constructed by sequentially combining the 21 elements for all of these properties and the 20 residues, resulting in a total of 188 dimensions.

The quasi-sequence order descriptors (Set D7) [96] are derived from both the Schneider-Wrede physicochemical distance matrix [10,18,97] and the Grantham chemical distance matrix [31], between each pair of the 20 amino acids. The physicochemical properties computed include hydrophobicity, hydrophilicity, polarity, and side-chain volume. Similar to the descriptors in Set D6, sequence order descriptors can also be used for representing amino acid distribution patterns of a specific physicochemical property along a protein or peptide sequence [18,31]. For a protein chain of *N *amino acid residues R_1_R_2_...R_*N*_, the sequence order effect can be approximately reflected through a set of sequence order coupling numbers

τd=∑i=1N−d(di,i+d)2,
 MathType@MTEF@5@5@+=feaafiart1ev1aaatCvAUfKttLearuWrP9MDH5MBPbIqV92AaeXatLxBI9gBaebbnrfifHhDYfgasaacH8akY=wiFfYdH8Gipec8Eeeu0xXdbba9frFj0=OqFfea0dXdd9vqai=hGuQ8kuc9pgc9s8qqaq=dirpe0xb9q8qiLsFr0=vr0=vr0dc8meaabaqaciaacaGaaeqabaqabeGadaaakeaaiiGacqWFepaDdaWgaaWcbaGaemizaqgabeaakiabg2da9maaqahabaGaeiikaGIaemizaq2aaSbaaSqaaiabdMgaPjabcYcaSiabdMgaPjabgUcaRiabdsgaKbqabaGccqGGPaqkdaahaaWcbeqaaiabikdaYaaaaeaacqWGPbqAcqGH9aqpcqaIXaqmaeaacqWGobGtcqGHsislcqWGKbaza0GaeyyeIuoakiabcYcaSaaa@44FB@

where τ_*d *_is the *d*th rank sequence order coupling number (*d *= 1, 2, ..., 30) that reflects the coupling mode between all of the most contiguous residues along a protein sequence, and *d*_*i*,*i*+*d *_is the distance between the two amino acids at position *i *and *i+d*. For each amino acid type, the type 1 quasi sequence order descriptor can be defined as

Xr=fr∑r=120fr+w∑d=130τd,
 MathType@MTEF@5@5@+=feaafiart1ev1aaatCvAUfKttLearuWrP9MDH5MBPbIqV92AaeXatLxBI9gBaebbnrfifHhDYfgasaacH8akY=wiFfYdH8Gipec8Eeeu0xXdbba9frFj0=OqFfea0dXdd9vqai=hGuQ8kuc9pgc9s8qqaq=dirpe0xb9q8qiLsFr0=vr0=vr0dc8meaabaqaciaacaGaaeqabaqabeGadaaakeaacqWGybawdaWgaaWcbaGaemOCaihabeaakiabg2da9maalaaabaGaemOzay2aaSbaaSqaaiabdkhaYbqabaaakeaadaaeWbqaaiabdAgaMnaaBaaaleaacqWGYbGCaeqaaOGaey4kaSIaem4DaC3aaabCaeaaiiGacqWFepaDdaWgaaWcbaGaemizaqgabeaaaeaacqWGKbazcqGH9aqpcqaIXaqmaeaacqaIZaWmcqaIWaama0GaeyyeIuoaaSqaaiabdkhaYjabg2da9iabigdaXaqaaiabikdaYiabicdaWaqdcqGHris5aaaakiabcYcaSaaa@4BFF@

where *r *= 1, 2, ..., 20, *f*_*r *_is the normalized occurrence of amino acid type *i *and *w *is a weighting factor (*w *= 0.1). The type 2 quasi sequence order is defined as

Xd=wτd−20∑r=120fr+w∑d=130τd,
 MathType@MTEF@5@5@+=feaafiart1ev1aaatCvAUfKttLearuWrP9MDH5MBPbIqV92AaeXatLxBI9gBaebbnrfifHhDYfgasaacH8akY=wiFfYdH8Gipec8Eeeu0xXdbba9frFj0=OqFfea0dXdd9vqai=hGuQ8kuc9pgc9s8qqaq=dirpe0xb9q8qiLsFr0=vr0=vr0dc8meaabaqaciaacaGaaeqabaqabeGadaaakeaacqWGybawdaWgaaWcbaGaemizaqgabeaakiabg2da9maalaaabaGaem4DaChcciGae8hXdq3aaSbaaSqaaiabdsgaKjabgkHiTiabikdaYiabicdaWaqabaaakeaadaaeWbqaaiabdAgaMnaaBaaaleaacqWGYbGCaeqaaOGaey4kaSIaem4DaC3aaabCaeaacqWFepaDdaWgaaWcbaGaemizaqgabeaaaeaacqWGKbazcqGH9aqpcqaIXaqmaeaacqaIZaWmcqaIWaama0GaeyyeIuoaaSqaaiabdkhaYjabg2da9iabigdaXaqaaiabikdaYiabicdaWaqdcqGHris5aaaakiabcYcaSaaa@5076@

where *d *= 21, 22, ..., 50. The combination of these two equations gives us a vector that describes a protein: the first 20 components reflect the effect of the amino acid composition, while the components from 21 to 50 reflect the effect of sequence order.

Similar to the quasi-sequence order descriptor, the pseudo amino acid descriptor (Set D8) is made up of a 50-dimensional vector in which the first 20 components reflect the effect of the amino acid composition and the remaining 30 components reflect the effect of sequence order, only now, the coupling number τ_*d *_is now replaced by the sequence order correlation factor θ_λ _[32]. The set of sequence order correlated factors is defined as follows:

θλ=1N−λ∑i=1L−λΘ(Ri,Ri+λ),
 MathType@MTEF@5@5@+=feaafiart1ev1aaatCvAUfKttLearuWrP9MDH5MBPbIqV92AaeXatLxBI9gBaebbnrfifHhDYfgasaacH8akY=wiFfYdH8Gipec8Eeeu0xXdbba9frFj0=OqFfea0dXdd9vqai=hGuQ8kuc9pgc9s8qqaq=dirpe0xb9q8qiLsFr0=vr0=vr0dc8meaabaqaciaacaGaaeqabaqabeGadaaakeaaiiGacqWF4oqCdaWgaaWcbaGae83UdWgabeaakiabg2da9maalaaabaGaeGymaedabaGaemOta4KaeyOeI0Iae83UdWgaamaaqahabaGaeuiMdeLaeiikaGIaemOuai1aaSbaaSqaaiabdMgaPbqabaGccqGGSaalcqWGsbGudaWgaaWcbaGaemyAaKMaey4kaSIae83UdWgabeaakiabcMcaPaWcbaGaemyAaKMaeyypa0JaeGymaedabaGaemitaWKaeyOeI0Iae83UdWganiabggHiLdGccqGGSaalaaa@4C65@

where θ_λ _is the first-tier correlation factor that reflects the sequence order correlation between all of the λ-most contiguous resides along a protein chain (λ = 1,...30) and *N *is the number of amino acid residues. Θ(R_*i*_, R_*j*_) is the correlation factor and is given by

Θ(Ri,Rj)=13{[H1(Rj)−H1(Ri)]2+[H2(Rj)−H2(Ri)]2+[M(Rj)−M(Ri)]2},
 MathType@MTEF@5@5@+=feaafiart1ev1aaatCvAUfKttLearuWrP9MDH5MBPbIqV92AaeXatLxBI9gBaebbnrfifHhDYfgasaacH8akY=wiFfYdH8Gipec8Eeeu0xXdbba9frFj0=OqFfea0dXdd9vqai=hGuQ8kuc9pgc9s8qqaq=dirpe0xb9q8qiLsFr0=vr0=vr0dc8meaabaqaciaacaGaaeqabaqabeGadaaakeaacqqHyoqucqGGOaakcqWGsbGudaWgaaWcbaGaemyAaKgabeaakiabcYcaSiabdkfasnaaBaaaleaacqWGQbGAaeqaaOGaeiykaKIaeyypa0ZaaSaaaeaacqaIXaqmaeaacqaIZaWmaaWaaiWaaeaadaWadaqaaiabdIeainaaBaaaleaacqaIXaqmaeqaaOGaeiikaGIaemOuai1aaSbaaSqaaiabdQgaQbqabaGccqGGPaqkcqGHsislcqWGibasdaWgaaWcbaGaeGymaedabeaakiabcIcaOiabdkfasnaaBaaaleaacqWGPbqAaeqaaOGaeiykaKcacaGLBbGaayzxaaWaaWbaaSqabeaacqaIYaGmaaGccqGHRaWkdaWadaqaaiabdIeainaaBaaaleaacqaIYaGmaeqaaOGaeiikaGIaemOuai1aaSbaaSqaaiabdQgaQbqabaGccqGGPaqkcqGHsislcqWGibasdaWgaaWcbaGaeGOmaidabeaakiabcIcaOiabdkfasnaaBaaaleaacqWGPbqAaeqaaOGaeiykaKcacaGLBbGaayzxaaWaaWbaaSqabeaacqaIYaGmaaGccqGHRaWkdaWadaqaaiabd2eanjabcIcaOiabdkfasnaaBaaaleaacqWGQbGAaeqaaOGaeiykaKIaeyOeI0Iaemyta0KaeiikaGIaemOuai1aaSbaaSqaaiabdMgaPbqabaGccqGGPaqkaiaawUfacaGLDbaadaahaaWcbeqaaiabikdaYaaaaOGaay5Eaiaaw2haaiabcYcaSaaa@7006@

where *H*_1_(R_*i*_), *H*_2_(R_*i*_) and *M*(R_*i*_) are the hydrophobicity [98], hydrophilicity [99] and side-chain mass of amino acid R_*i*_, respectively. Before being substituted in the above equation, the various physicochemical properties *P*(*i*) are subjected to a standard conversion,

P(i)=P0(i)−∑i=120P0(i)20∑i=120[P0(i)−∑i=120P0(i)20]220
 MathType@MTEF@5@5@+=feaafiart1ev1aaatCvAUfKttLearuWrP9MDH5MBPbIqV92AaeXatLxBI9gBaebbnrfifHhDYfgasaacH8akY=wiFfYdH8Gipec8Eeeu0xXdbba9frFj0=OqFfea0dXdd9vqai=hGuQ8kuc9pgc9s8qqaq=dirpe0xb9q8qiLsFr0=vr0=vr0dc8meaabaqaciaacaGaaeqabaqabeGadaaakeaacqWGqbaucqGGOaakcqWGPbqAcqGGPaqkcqGH9aqpdaWcaaqaaiabdcfaqnaaCaaaleqabaGaeGimaadaaOGaeiikaGIaemyAaKMaeiykaKIaeyOeI0YaaabCaeaadaWcaaqaaiabdcfaqnaaCaaaleqabaGaeGimaadaaOGaeiikaGIaemyAaKMaeiykaKcabaGaeGOmaiJaeGimaadaaaWcbaGaemyAaKMaeyypa0JaeGymaedabaGaeGOmaiJaeGimaadaniabggHiLdaakeaadaGcaaqaamaalaaabaWaaabCaeaadaWadaqaaiabdcfaqnaaCaaaleqabaGaeGimaadaaOGaeiikaGIaemyAaKMaeiykaKIaeyOeI0YaaabCaeaadaWcaaqaaiabdcfaqnaaCaaaleqabaGaeGimaadaaOGaeiikaGIaemyAaKMaeiykaKcabaGaeGOmaiJaeGimaadaaaWcbaGaemyAaKMaeyypa0JaeGymaedabaGaeGOmaiJaeGimaadaniabggHiLdaakiaawUfacaGLDbaaaSqaaiabdMgaPjabg2da9iabigdaXaqaaiabikdaYiabicdaWaqdcqGHris5aOWaaWbaaSqabeaacqaIYaGmaaaakeaacqaIYaGmcqaIWaamaaaaleqaaaaaaaa@68BB@

This sequence order correlation definition [Eqs. (14), (15)] introduce more correlation factors of physicochemical effects as compared to the coupling number [Eq. (11)], and has shown to be an improvement on the way sequence order effect information is represented [32,35,100]. Thus, for each amino acid type, the first part of the vector is defined as

Xr=fr∑r=120fr+w∑d=130θj,
 MathType@MTEF@5@5@+=feaafiart1ev1aaatCvAUfKttLearuWrP9MDH5MBPbIqV92AaeXatLxBI9gBaebbnrfifHhDYfgasaacH8akY=wiFfYdH8Gipec8Eeeu0xXdbba9frFj0=OqFfea0dXdd9vqai=hGuQ8kuc9pgc9s8qqaq=dirpe0xb9q8qiLsFr0=vr0=vr0dc8meaabaqaciaacaGaaeqabaqabeGadaaakeaacqWGybawdaWgaaWcbaGaemOCaihabeaakiabg2da9maalaaabaGaemOzay2aaSbaaSqaaiabdkhaYbqabaaakeaadaaeWbqaaiabdAgaMnaaBaaaleaacqWGYbGCaeqaaOGaey4kaSIaem4DaC3aaabCaeaaiiGacqWF4oqCdaWgaaWcbaGaemOAaOgabeaaaeaacqWGKbazcqGH9aqpcqaIXaqmaeaacqaIZaWmcqaIWaama0GaeyyeIuoaaSqaaiabdkhaYjabg2da9iabigdaXaqaaiabikdaYiabicdaWaqdcqGHris5aaaakiabcYcaSaaa@4BFC@

where *r *= 1, 2, ..., 20, *f*_*r *_is the normalized occurrence of amino acid type *i *and *w *is a weighting factor (*w *= 0.1), and the second part is defined as

Xd=wθd−20∑r=120fr+w∑d=130ϑλ.
 MathType@MTEF@5@5@+=feaafiart1ev1aaatCvAUfKttLearuWrP9MDH5MBPbIqV92AaeXatLxBI9gBaebbnrfifHhDYfgasaacH8akY=wiFfYdH8Gipec8Eeeu0xXdbba9frFj0=OqFfea0dXdd9vqai=hGuQ8kuc9pgc9s8qqaq=dirpe0xb9q8qiLsFr0=vr0=vr0dc8meaabaqaciaacaGaaeqabaqabeGadaaakeaacqWGybawdaWgaaWcbaGaemizaqgabeaakiabg2da9maalaaabaGaem4DaChcciGae8hUde3aaSbaaSqaaiabdsgaKjabgkHiTiabikdaYiabicdaWaqabaaakeaadaaeWbqaaiabdAgaMnaaBaaaleaacqWGYbGCaeqaaOGaey4kaSIaem4DaC3aaabCaeaacqWFrpGsdaWgaaWcbaGae83UdWgabeaaaeaacqWGKbazcqGH9aqpcqaIXaqmaeaacqaIZaWmcqaIWaama0GaeyyeIuoaaSqaaiabdkhaYjabg2da9iabigdaXaqaaiabikdaYiabicdaWaqdcqGHris5aaaakiabc6caUaaa@50AC@

### Support Vector Machines (SVM)

As the SVM algorithms have been extensively described in the literature [2,3,101], only a brief description is given here. In the case of a linear SVM, a hyperplane that separates two different classes of feature vectors with a maximum margin is constructed. One class represents positive samples, for example EC2.4 proteins, and the other the negative samples. This hyperplane is constructed by finding a vector **w **and a parameter *b *that minimizes ||**w**||^2 ^that satisfies the following conditions: **w**·**x**_*i *_+ *b *≥ 1, for *y*_*i *_= 1 (positive class) and **w**·**x**_*i *_+ *b *≤ -1, for *y*_*i *_= -1 (negative class). Here **x**_*i *_is a feature vector, *y*_*i *_is the group index, **w **is a vector normal to the hyperplane, |b|||w||
 MathType@MTEF@5@5@+=feaafiart1ev1aaatCvAUfKttLearuWrP9MDH5MBPbIqV92AaeXatLxBI9gBaebbnrfifHhDYfgasaacH8akY=wiFfYdH8Gipec8Eeeu0xXdbba9frFj0=OqFfea0dXdd9vqai=hGuQ8kuc9pgc9s8qqaq=dirpe0xb9q8qiLsFr0=vr0=vr0dc8meaabaqaciaacaGaaeqabaqabeGadaaakeaadaWcaaqaaiabcYha8jabdkgaIjabcYha8bqaaiabcYha8jabcYha8jabhEha3jabcYha8jabcYha8baaaaa@3884@ is the perpendicular distance from the hyperplane to the origin, and ||**w**||^2 ^is the Euclidean norm of **w**. In the case of a nonlinear SVM, feature vectors are projected into a high dimensional feature space by using a kernel function such as K(xi,xj)=e−‖xi−xj‖2/2σ2
 MathType@MTEF@5@5@+=feaafiart1ev1aaatCvAUfKttLearuWrP9MDH5MBPbIqV92AaeXatLxBI9gBaebbnrfifHhDYfgasaacH8akY=wiFfYdH8Gipec8Eeeu0xXdbba9frFj0=OqFfea0dXdd9vqai=hGuQ8kuc9pgc9s8qqaq=dirpe0xb9q8qiLsFr0=vr0=vr0dc8meaabaqaciaacaGaaeqabaqabeGadaaakeaacqWGlbWscqGGOaakcqWH4baEdaWgaaWcbaGaemyAaKgabeaakiabcYcaSiabhIha4naaBaaaleaacqWGQbGAaeqaaOGaeiykaKIaeyypa0Jaemyzau2aaWbaaSqabeaacqGHsisldaqbdaqaaiabhIha4naaBaaameaacqWGPbqAaeqaaSGaeyOeI0IaeCiEaG3aaSbaaWqaaiabdQgaQbqabaaaliaawMa7caGLkWoadaahaaadbeqaaiabikdaYaaaliabc+caViabikdaYGGaciab=n8aZnaaCaaameqabaGaeGOmaidaaaaaaaa@4A11@. The linear SVM procedure is then applied to the feature vectors in this feature space. After the determination of **w **and *b*, a given vector **x **can be classified by using *sign *[(**w**.**x**) + *b*], a positive or negative value indicating that the vector **x **belongs to the positive or negative class respectively.

As a discriminative method, the performance of SVM classification can be accessed by measuring the true positive *TP *(correctly predicted positive samples), false negative *FN *(positive samples incorrectly predicted as negative), true negative *TN *(correctly predicted negative samples), and false positive *FP *(negative samples incorrectly predicted as positive) [4,102,103]. As the numbers of positive and negative samples are imbalanced, the positive prediction accuracy or sensitivity *Q*_*p *_= *TP*/(*TP+FN*) and negative prediction accuracy or specificity *Q*_*n *_= *TN*/(*TN+FP*) [101] are also introduced. The overall accuracy is defined as *Q *= (*TP+TN*)/(*TP+FN+TN+FP*). However, in some cases, *Q*, *Q*_*p*_, and *Q*_*n*_are insufficient to provide a complete assessment of the performance of a discriminative method [102,104]. Thus the Matthews correlation coefficient (MCC) was used in this work to evaluate the randomness of the prediction:

MCC=TP×TN−FP×FN(TP+FN)(TP+FP)(TN+FP)(TN+FN),
 MathType@MTEF@5@5@+=feaafiart1ev1aaatCvAUfKttLearuWrP9MDH5MBPbIqV92AaeXatLxBI9gBaebbnrfifHhDYfgasaacH8akY=wiFfYdH8Gipec8Eeeu0xXdbba9frFj0=OqFfea0dXdd9vqai=hGuQ8kuc9pgc9s8qqaq=dirpe0xb9q8qiLsFr0=vr0=vr0dc8meaabaqaciaacaGaaeqabaqabeGadaaakeaacqWGnbqtcqWGdbWqcqWGdbWqcqGH9aqpdaWcaaqaaiabdsfaujabdcfaqjabgEna0kabdsfaujabd6eaojabgkHiTiabdAeagjabdcfaqjabgEna0kabdAeagjabd6eaobqaamaakaaabaGaeiikaGIaemivaqLaemiuaaLaey4kaSIaemOrayKaemOta4KaeiykaKIaeiikaGIaemivaqLaemiuaaLaey4kaSIaemOrayKaemiuaaLaeiykaKIaeiikaGIaemivaqLaemOta4Kaey4kaSIaemOrayKaemiuaaLaeiykaKIaeiikaGIaemivaqLaemOta4Kaey4kaSIaemOrayKaemOta4KaeiykaKcaleqaaaaakiabcYcaSaaa@5CEB@

where MCC ∈ [-1,1], with a negative value indicating disagreement of the prediction and a positive value indicating agreement. A zero value means the prediction is completely random. The MCC utilizes all four basic elements of the accuracy and it provides a better summary of the prediction performance than the overall accuracy.

## Authors' contributions

SAK generated the datasets, carried out the calculations and drafted the manuscript, HH generated the datasets and participated in the design of the study, ZR updated the descriptor generation program to calculate PseAA descriptors (ZR wrote the original descriptor generation program, introduced in previous works), YZ conceived of the study and corrected the manuscript, and YZ and ZW oversaw the design and coordination of this work and provided invaluable advice. All authors read and approved the final manuscript.

## References

[B1] Karchin R, Karplus K, Haussler D (2002). Classifying G-protein coupled receptors with support vector machines. Bioinformatics.

[B2] Cai CZ, Han LY, Ji ZL, Chen X, Chen YZ (2003). SVM-Prot: web-based support vector machine software for functional classification of a protein from its primary sequence. Nuclei Acid Res.

[B3] Cai CZ, Han LY, Ji ZL, Chen YZ (2004). Enzyme family classification by support vector machines. Proteins.

[B4] Han LY, Cai CZ, Lo SL, Chung MC, Chen YZ (2004). Prediction of RNA-binding proteins from primary sequence by a support vector machine approach. RNA.

[B5] Dubchak I, Mayor C, Dralyuk I, Kim SH, Muchnick I (1999). Recognition of a protein fold in the context of the Structural Classification of Proteins (SCOP) classification. Proteins.

[B6] Bock JR, Gough DA (2001). Predicting protein--protein interactions from primary structure. Bioinformatics.

[B7] Bock JR, Gough DA (2003). Whole-proteome interaction mining. Bioinformatics.

[B8] Lo SL, Cai CZ, Chen YZ, Chung MC (2005). Effect of training datasets on support vector machine prediction of protein-protein interactions. Proteomics.

[B9] Chou KC, Cai YD (2006). Predicting protein-protein interactions from sequences in a hybridization space. J Proteome Res.

[B10] Chou KC (2000). Prediction of protein subcellular locations by incorporating quasi-sequence-order effect. Biochem Biophys Res Commun.

[B11] Chou KC, Cai YD (2004). Prediction of protein subcellular locations by GO-FunD-PseAA predictor. Biochem Biophys Res Commun.

[B12] Chou KC, Shen HB (2006). Hum-PLoc: A novel ensemble classifier for predicting human protein subcellular localization. Biochem Biophys Res Commun.

[B13] Chou KC, Shen HB (2006). Large-scale plant protein subcellular location prediction. J Cell Biochem.

[B14] Bhasin M, Garg A, Raghava GP (2005). PSLpred: prediction of subcellular localization of bacterial proteins. Bioinformatics.

[B15] Guo J, Lin Y, Liu XJ (2006). GNBSL: a new integrative system to predict the subcellular location for Gram-negative bacteria proteins. Proteomics.

[B16] Guo J, Lin Y (2006). TSSub: eukaryotic protein subcellular localization by extracting features from profiles. Bioinformatics.

[B17] Cui J, Han LY, Lin HH, Zhang HL, Tang ZQ, Zheng CJ, Cao ZW, Chen YZ (2007). Prediction of MHC-binding peptides of flexible lengths from sequence-derived structural and physicochemical properties. Mol Immunol.

[B18] Schneider G, Wrede P (1994). The rational design of amino acid sequences by artificial neural networks and simulated molecular evolution: de novo design of an idealized leader peptidase cleavage site. Biophys J.

[B19] Brown MP, Grundy WN, Lin D, Cristianini N, Sugnet CW, Furey TS, Ares MJ, Haussler D (2000). Knowledge-based analysis of microarray gene expression data by using support vector machines. Proc Natl Acad Sci USA.

[B20] Ward JJ, McGuffin LJ, Buxton BF, Jones DT (2003). Secondary structure prediction with support vector machines. Bioinformatics.

[B21] Han LY, Cai CZ, Ji ZL, Cao ZW, Cui J, Chen YZ (2004). Predicting functional family of novel enzymes irrespective of sequence similarity: a statistical learning approach. Nuclei Acid Res.

[B22] Li ZR, Lin HH, Han LY, Jiang L, Chen X, Chen YZ (2006). PROFEAT: A web server for computing structural and physicochemical features of proteins and peptides from amino acid sequence. Nuclei Acid Res.

[B23] Chou KC, Cai YD (2005). Prediction of membrane protein types by incorporating amphipathic effects. J Chem Inf Model.

[B24] Gao QB, Wang ZZ, Yan C, Du YH (2005). Prediction of protein subcellular location using a combined feature of sequence.. FEBS Lett.

[B25] Feng ZP, Zhang CT (2000). Prediction of membrane protein types based on the hydrophobic index of amino acids. J Protein Chem.

[B26] Lin Z, Pan XM (2001). Accurate prediction of protein secondary structural content. J Protein Chem.

[B27] Horne DS (1988). Prediction of protein helix content from an autocorrelation analysis of sequence hydrophobicities. Biopolymers.

[B28] Sokal RR, Thomson BA (2006). Population structure inferred by local spatial autocorrelation: an example from an Amerindian tribal population. Am J Phys Anthropol.

[B29] Dubchak I, I M, Holbrook SR, Kim SH (1995). Prediction of protein folding class using global description of amino acid sequence. Proc Natl Acad Sci USA.

[B30] Lin HH, Han LY, Cai CZ, Ji ZL, Chen YZ (2006). Prediction of transporter family from protein sequence by support vector machine approach. Proteins.

[B31] Grantham R (1974). Amino acid difference formula to help explain protein evolution. Science.

[B32] Chou KC (2001). Prediction of protein cellular attributes using pseudo amino acid composition. Proteins: Structure Function and Genetics.

[B33] Bhasin M, Raghava GP (2004). Classification of nuclear receptors based on amino acid composition and dipeptide composition. J Biol Chem.

[B34] NC-IUBMB (1992). Enzyme Nomenclature.

[B35] Chou KC (2005). Using amphiphilic pseudo amino acid composition to predict enzyme subfamily classes. Bioinformatics.

[B36] Chou KC, Cai YD (2004). Predicting enzyme family class in a hybridization space. Protein Sci.

[B37] Chou KC, Elrod DW (2003). Prediction of enzyme family classes. J Proteome Res.

[B38] Chou KC (2005). Prediction of G-protein-coupled receptor classes. J Proteome Res.

[B39] Chou KC, Elrod DW (2002). Bioinformatical analysis of G-protein-coupled receptors. J Proteome Res.

[B40] Bhasin M, Raghava GP (2004). GPCRpred: an SVM-based method for prediction of families and subfamilies of G-protein coupled receptors. Nuclei Acid Res.

[B41] Saier MHJ, Tran CV, Barabote RD (2006). TCDB: the Transporter Classification Database for membrane transport protein analyses and information. Nuclei Acid Res.

[B42] Suzuki JY, Bollivar DW, Bauer CE (1997). Genetic analysis of chlorophyll biosynthesis. Annu Rev Genet.

[B43] Lin HH, Han LY, Zhang HL, Zheng CJ, Xie B, Chen YZ (2006). Prediction of the functional class of lipid binding proteins from sequence-derived properties irrespective of sequence similarity. J Lipid Res.

[B44] Brown MP, Grundy WN, Lin D, Cristianini N, Sugnet CW, Furey TS, Ares MJ, Haussler D (2000). Knowledge-based analysis of microarray gene expression data by using support vector machines.. Proc Natl Acad Sci USA.

[B45] Burbidge R, Trotter M, Buxton B, Holden S (2001). Drug design by machine learning: support vector machines for pharmaceutical data analysis. Comput Chem.

[B46] Baenzigner JU (1994). Protein-specific glycosyltransferase: how and why they do it!. FASEB J.

[B47] Kapitonov D, Yu RK (1999). Conserved domains of glycosyltransferase. Glycobiology.

[B48] Busch W, Saier MHJ (2002). The Transporter Classification (TC) system. Crit Rev Biochem Mol Biol.

[B49] Drews J (1996). Genomic sciences and the medicine of tomorrow. Nat Biotechnol.

[B50] Gudermann TB, Nurnberg B, Schultz G (1995). Receptors and G proteins as primary components of transmembrane signal transduction. Part 1. G-protein-coupled receptors: structure and function. J Mol Med.

[B51] Muller G (2000). Towards 3D structures of G protein-coupled receptors: a multidisciplinary approach. Curr Med Chem.

[B52] Paulson JC, Colley KJ (1989). Glycosyltransferase. J Biol Chem.

[B53] Beale SI, Weinstein JD, Jordan PM (1991). Biochemistry and regulation of photosynthetic pigment formation in plants and algae. Biosynthesis of Tetrapyrroles.

[B54] Glatz JF, Luiken JJ, van Bilsen M, van der Vusse GJ (2002). Cellular lipid binding proteins as facilitators and regulators of lipid metabolism.. Mol Cell Biochem.

[B55] Burd CG, Dreyfuss G (1994). Conserved structures and diversity of functions of RNA-binding proteins. Science.

[B56] Kiledjian M, Burd CG, Portman DS, Gorlach M, Dreyfuss G, Nagai K, Mattaj IW (1994). Structure and function of hnRNP proteins. RNA-Protein Interactions: Frontiers in Molecular Biology.

[B57] Draper DE (1999). Themes in RNA-protein recognition. J Mol Biol.

[B58] Fierro-Monti I, Mathews MB (2000). Proteins binding to duplexed RNA: one motif, multiple functions. Trends Biochem Sci.

[B59] Perculis BA (2000). RNA-binding proteins: If it looks like a sn(o)RNA. Curr Biol.

[B60] Perez-Canadillas JM, Varani G (2001). Recent advances in RNA-protein recognition. Curr Opin Struct Biol.

[B61] Chou KC, Zhang CT (1995). Prediction of protein structural classes. Crit Rev Biochem Mol Biol.

[B62] Li WZ, Godzik A (2006). Cd-hit: a fast program for clustering and comparing large sets of proteins or nucleotide sequences. Bioinformatics.

[B63] Li WZ, Jaroszewksi L, Godzik A (2001). Clustering of highly homologous sequences to reduce the size of large protein database. Bioinformatics.

[B64] Li WZ, Jaroszewksi L, Godzik A (2002). Tolerating some redundancy significantly speeds up clustering of large protein databases. Bioinformatics.

[B65] Garg A, Bhasin M, Raghava GP (2005). Support vector machine-based method for subcellular localization of human proteins using amino acid compositions, their order, and similarity search. J Biol Chem.

[B66] Bhasin M, Raghava GP (2004). ESLpred: SVM-based method for subcellular localization of eukaryotic proteins using dipeptide composition and PSI-BLAST. Nuclei Acid Res.

[B67] Xue L, Bajorath J (2000). Molecular descriptors in chemoinformatics, computational combinatorial chemistry, and virtual screening. Comb Chem High Throughput Screen.

[B68] Xue L, Godden JW, Bajorath J (1999). Identification of a preferred set of descriptors for compound classification based on principal component analysis. J Chem Inf Comput Sci.

[B69] Xue Y, Li ZR, Yan CW, Sun LZ, Chen X, Chen YZ (2004). Effect of molecular descriptor feature selection in support vector machine classification of pharmacokinetic and toxicological properties of chemical agents.. J Chem Inf Comput Sci.

[B70] Brown RD, Martin YC (1996). Use of structure-activity data to compare structure-based clustering methods and descriptors for use in compound selection. J Chem Inf Comput Sci.

[B71] Cramer RD, Patterson DE, Bunce JD (1988). Comparative molecular field analysis (CoMFA): effect of shape on binding of steroids to carrier proteins. J Am Chem Soc.

[B72] Glen WG, Dunn WJ, Scott RD (1989). Principal components analysis and partial least squares regression. Tetrahedron Comput Methodol.

[B73] Matter H (1997). Selecting optimally diverse compounds from structure databases: a validation study of two-dimensional and three-dimensional molecular descriptors. J Med Chem.

[B74] Matter H, Pötter T (1999). Comparing 3D pharmacophore triplets and 2D fingerprints for selecting diverse compound subsets. J Chem Inf Comput Sci.

[B75] Patterson DEP, Cramer RD, Ferguson AM, Clark RD, Weinberger LE (1996). Neighborhood behavior: a useful concept for validation of "molecular diversity" descriptors. J Med Chem.

[B76] Xue L, Godden JW, Bajorath J (2000). Evaluation of descriptors and mini-fingerprints for the identification of molecules with similar activity.. J Chem Inf Comput Sci.

[B77] Lin HH, Han LY, Zhang HL, Zheng CJ, Xie B, Chen YZ (2006). Prediction of the functional class of DNA-binding proteins from sequence derived structural and physicochemical properties.

[B78] Chen C, Zhou X, Tian Y, Zhou X, Cai P (2006). Predicting protein structural class with pseudo-amino acid composition and support vector machine fusion network. Anal Biochem.

[B79] Furey TS, Cristianini N, Duffy N, Bednarski DW, Schummer M, Haussler D (2000). Support vector machines classification and validation of cancer tissue samples using microarray expression data.. Bioinformatics.

[B80] Yu H, Yang J, Wang W, Han J (2003). Discovering compact and highly discriminative features or feature combinations of drug activities using support vector machines.. Proc IEEE Comput Soc Bioinform Conf.

[B81] Boeckmann B, Bairoch A, Apweiler R, Blatter MC, Estreicher A, Gasteiger E, Martin MJ, Michoud K, O'Donovan C, Phan I, Pilbout S, Schneider M (2003). The SWISS-PROT protein knowledgebase and its supplement TrEMBL in 2003. Nuclei Acid Res.

[B82] Bateman A, Birney E, Cerruti L, Durbin R, Etwiller L, Eddy SR, Griffiths–Jones S, Howe KL, Marshall M, Sonnhammer EL (2002). The Pfam protein families database. Nuclei Acid Res.

[B83] Heyer LJ, Kruglyak S, Yooseph S (1999). Exploring expression data: Identification and analysis of coexpressed genes. Genome Res.

[B84] Broto P, Moreau G, Vandicke C (1984). Molecular structures: perception, autocorrelation descriptor and SAR studies. Eur J Med Chem.

[B85] Kawashima S, Kanehisa M (2000). AAindex: amino acid index database. Nuclei Acid Res.

[B86] Cid H, Bunster M, Canales M, Gazitua F (1992). Hydrophobicity and structural classes in proteins. Protein Eng.

[B87] Bhaskaran R, Ponnuswammy PK (1988). Positional flexibilities of amino acid residues in globular proteins. Int J Pept Protein Res.

[B88] Charton M, Charton BI (1982). The structural dependence of amino acid hydrophobicity parameters. J Theor Biol.

[B89] Chothia C (1976). The nature of the accessible and buried surfaces in proteins. J Mol Biol.

[B90] Bigelow CC (1967). On the average hydrophobicity of proteins and the relation between it and protein structure. J Theor Biol.

[B91] Charton M (1981). Protein folding and the genetic code: an alternative quantitative model. J Theor Biol.

[B92] Dayhoff H, Calderone H (1978). Composition of proteins. Atlas of Protein Sequence and Structure.

[B93] Moreau G, Broto P (1980). Autocorrelation of molecular structures, application to SAR studies. Nour J Chim.

[B94] Moran PAP (1950). Notes on continuous stochastic phenomena. Biometrika.

[B95] Geary RC (1954). The contiguity ratio and statistical mapping. Incorp Statist.

[B96] Cai YD, Liu XJ, Xu X, Chou KC (2002). Support vector machines for prediction of protein subcellular location by incorporating quasi-sequence-order effect. J Cell Biochem.

[B97] Chou KC, Cai YD (2002). Using functional domain composition and support vector machines for prediction of protein subcellular location. J Biol Chem.

[B98] Jones DD (1975). Amino acid properties and side-chain orientation in proteins: a cross correlation approach.. J Theor Biol.

[B99] Hopp TP, Woods KR (1981). Prediction of protein antigenic determinants from amino acid sequences.. Proc Natl Acad Sci USA.

[B100] Feng ZP (2002). An overview on predicting the subcellular location of a protein. In Silico Biol.

[B101] Burges CJC (1998). A tutorial on support vector machines for pattern recognition. Data Min Knowl Dis.

[B102] Baldi P, Brunak S, Chauvin Y, Andersen CA, Nielsen H (2000). Assessing the accuracy of prediction algorithms for classification: an overview. Bioinformatics.

[B103] Roulston JE (2002). Screening with tumor markers: critical issues. Mol Biotechnol.

[B104] Provost F, Fawcett T, Kohavi R (1998). The case against accuracy estimation for comparing induction algorithms. Proc 15th International Conf on Machine Learning.

